# Understanding Family Migration in Rural South Africa: Exploring Children's Inclusion in the Destination Households of Migrant Parents

**DOI:** 10.1002/psp.1842

**Published:** 2014-01-08

**Authors:** Rachel Bennett, Victoria Hosegood, Marie-Louise Newell, Nuala McGrath

**Affiliations:** 1 University of SouthamptonUK; 2 Africa Centre for Health and Population Studies, University of KwaZulu-NatalSouth Africa

**Keywords:** children, migrant parents, family migration, destination household, South Africa

## Abstract

Despite the removal of restrictions on movement and increasing female participation in migration, only a minority of migrant parents in South Africa include their children in their destination household. Quantitative analyses of the circumstances in which children accompany a migrant parent have been limited by the lack of available data that document family arrangements from the perspective of more than one household. This paper uses data about members of rural households in a demographic surveillance population in KwaZulu-Natal and a linked sample survey of adult migrants to examine factors associated with children's inclusion in the destination household of migrant parents, analyse the timing and sequence of children's moves to parental destination households, and describe the composition of parental origin and destination households. The findings confirm that in contemporary South Africa, only a small percentage (14%) of migrants' children who are members of the parental origin household are also members of the parental destination household. Membership of the parental destination household is associated with parental characteristics and the child's age, but not measures of socio-economic status, and children most commonly migrate several years *after* their migrant parent. Children included in the destination household of migrant fathers frequently live in small households, which also include their mother, whereas children included in the destination household of migrant mothers live in larger households. This study contributes to understanding the contexts of children's inclusion in parental destination households in South Africa and demonstrates the potential of data collected in migrants' origin and destination households.

## Introduction

Circular adult labour migration has been deeply entrenched in South Africa's social system since the early 20th century. In contemporary rural South Africa, despite political reforms and increases in female participation in the labour market, recent national surveys suggest only a minority of migrant parents include their children in their destination households (Posel, 2010). Recent calls to support families to promote the health and well-being of children in South Africa have drawn attention to the need for further evidence on the spatial distribution of children in relation to parents and family members (Sherr *et al*., 2008; Hosegood & Madhavan, 2010). Analyses of the circumstances in which migrants include their children in their destination household are important for understanding the family and care arrangements of migrants' children and contribute to this evidence base (Hall & Posel, 2012). In this paper, we use surveillance data from the Africa Centre Demographic Information System (ACDIS) in rural KwaZulu-Natal and a nested sample of migrants, the non-residents living arrangements (NRLA) survey, to explore the contexts of children's inclusion in migrant parents' destination households. The objectives of the paper are (i) to identify factors associated with children's inclusion in the parental destination household, (ii) to analyse the timing and sequence of children's moves to parental destination households in relation to their migrant parent, and (iii) to describe the composition and characteristics of the origin and destination households of migrant parents. The paper begins with an overview of studies of family migration in South Africa and the decision about whether children are included in destination households of migrant parents. The subsequent sections describe the data and methodological approach and present the results. The final section draws conclusions about the contexts of children's inclusion in the destination households of migrant parents in South Africa.

## Parental Migration and Children's Living Arrangements in South Africa

In the colonial and apartheid eras, government policy highly regulated the movement and settlement of non-white population groups. The 10 tribal self-governing homelands (*Bantustans*) located in rural often remote areas of the country provided very few employment opportunities, and therefore, black African men and women migrated to work in cities, towns, and commercial farms. Permanent family migration was inhibited by legislation designed to control settlement in urban areas; therefore, labour migration patterns were predominately circular (Jones, 1993; Moser, 1999). Migrants would retain social ties to their origin rural households through regular visits or return migration. Children of migrants typically remained resident in the origin community in the care of other family members.

In contemporary rural South Africa, levels of temporary adult migration remain high. Two longitudinal population-based studies conducted in different parts of South Africa use a definition of household membership based on social connection rather than residency. Collinson (2009) found 41% of adult male (15 years or older) and 18% of adult female household members in the Agincourt demographic surveillance system in the Mpumalanga province spent at least 6 months away from their households in 2007. Similarly, Muhwava *et al*. (2010) analysing data from the Africa Centre demographic surveillance system in KwaZulu-Natal showed that in mid-2008, 38% of adult male (18 years or older) and 32% of adult female household members were residing outside the study area. In addition to employment and work seeking, other reasons for adult migration include marriage and partnership formation and dissolution, housing, education, and training. An increasing proportion of migrants are female, linked to the removal of restrictions on movement and simultaneous decline in marriage rates and female co-residence with men and increase in female labour force participation. Results from national household surveys conducted in 1993 and 1999 found evidence of an increase in temporary labour migration overall in this period, which was largely attributed to an increase in female migration (Posel, 2003). In 1993, 30% of migrant workers were women, whereas by 1999, this had increased to approximately 34% (Posel, 2003). In the context of rural KwaZulu-Natal, Camlin (2008) analysing data from the Africa Centre demographic surveillance system between 2000 and 2003 found a higher proportion of women than men (16.7% vs 13%) were engaged in local moves. Female migrants frequently maintain stronger ties with their origin household and are more likely to be driven by extreme poverty than their male counterparts (Collinson, 2009).

Recent studies using data collected from respondents in rural households suggest that most parents that migrate do not bring their children to live with them in their destination household, and instead, children are left in the care of other family members. Kautzky (2009) used cross-sectional survey data collected in 2007 in the Agincourt sub-district of the Mpumalanga Province to examine migrant parents' choices about their children's living arrangements. The study found that only 11% of parents included at least one child in their destination household, higher amongst mothers than fathers (14% compared with 10%, *p* < 0.01), a finding consistent with the higher rates of co-residence between mothers and their children than fathers and their children overall in South Africa (Meintjes & Hall, 2012).

Work by Russell (2003) on black urban households in South Africa shows that rural areas are often seen as preferred places for children to grow up. Destination households of parents may be crowded, and depending on the strength of the connection between migrant parents and other household members, they may not feel able to bring family members (Smit, 1998; Fall, 1998). Studies have also documented poor material conditions for migrant families in their destination community. Richter *et al*. (2006) found that children who migrated to Johannesburg were more likely to live in informal housing (a shack, garage, or cottage) than formal housing and were less likely to have good access to electricity, refuse removal, water, and sanitation than long-standing residents.

The existence and quality of family connections between origin and destination households are important influences on the decision by parents and other family members about where children should live. However, the connections between migrant parents' origin and destination households do not necessarily remain constant over time. For example, after becoming well-established in their destination community, a migrant parent's perception of ‘home’ may change. Richter *et al*. (2006, p. 10) describe ‘a gradient’ of quality of access to housing and services that frequently improves with length of migration. Gilbert and Crankshaw (1999) analysed data from a survey in Soweto, Johannesburg, and suggested that if migrants become well established in the destination area, they are more likely to have their immediate families with them and less likely to maintain strong links with origin households. However, it may also be a long-term conscious choice for children not to join their parents in the destination community. Ngwane (2003, p. 689) has described the way in which migration may be an ‘alternative means of being local’ for some parents by enabling them to financially support their families without having to move dependents from the family base. Smit (1998) has suggested that a continuum of ‘relatedness’ exists between migrants and their destination and origin households. This continuum ranges from migrants who perceive their origin household to be their true family home and will typically have a single defined purpose for their presence in their destination community, such as to earn money or to further their education, to migrants who see themselves and their current lives as completely separate from their origin household.

Household-based surveys and studies seldom collect data about or from the destination households to which members have migrated. This has limited the ability of empirical studies to directly compare the circumstances of children who do and do not live with their migrant parent in their destination household. In this study, we use new survey and surveillance data to explore the contexts of children's inclusion in migrant parents' destination households from the perspective of parental origin and destination households. The following section provides a brief overview of the data and statistical methods used for the analyses in this paper. A detailed description of the data sources and methodology for analysing data collected in migrants' origin and destination households, as well as the application of the data to examining the circumstances of children ‘left behind’ by migrant parents, is provided in a companion paper published in this issue (Bennett *et al*., 2014).

## Data and Methods

### Africa Centre Demographic Information System (ACDIS)

The ACDIS has been in operation since 2000 and contains detailed socio-demographic longitudinal data about the whole population of a predominately rural 438 km^2^ demographic surveillance area (DSA) in northern KwaZulu-Natal (Tanser *et al*., 2008). Data are collected two (until 2012) or three (since 2012) times a year every year, and each round includes approximately 90,000 members of the 11,000 households in the study area (Tanser *et al*., 2008). The average household size is 7.9 members, and the primary sources of income for most households are state pensions and/or waged employment. Approximately 7% of the mid-year population migrates annually, predominately within the province of KwaZulu-Natal (Muhwava *et al*., 2010). For both men and women, the main self-reported reasons for migration are accommodation, employment, and education (Muhwava *et al*., 2010).

The ACDIS includes all households living in a bounded structure (homestead) in the DSA, and all individual household members are recorded. Household membership is defined by respondents and primarily relates to perceptions of social connectedness and belonging. An individual will be recorded as a *resident* household member if they usually sleep in the household and keep their belongings there. An individual will be recorded as a *non-resident* household member if they do not fulfil these conditions but remain socially connected to the household as a social group. This broadly relates to the inclusion criteria increasingly used in household surveys, such as those conducted under the World Bank's Living Standards Measurement Study, which seek to collect information on individuals who currently live in a household plus individuals who fail to meet residency criteria but are still considered to belong to the household (Carletto & de Brauw, 2007). An individual is recorded to have out-migrated when they end a period of residency with a household. They will then be recorded as a non-resident household member if they are perceived by the respondent to retain social ties with the household. Information is collected on all resident and non-resident household members and includes data on births, deaths, migrations, marriages, parental survival, and individual and household socio-economic status.

### Non-Residents Living Arrangements (NRLA) Survey

Since 2003, annual HIV surveillance has been conducted with a stratified sample of non-resident members of households in the DSA (women aged 15–49 years and men aged 15–54 years), who are living in households outside the DSA. In 2009, the NRLA survey was added as a cross-sectional module to the questionnaire administered to the sample of non-resident members of the ACDIS (McGrath *et al*., 2008). The survey included a complete household roster, which makes it suitable for examining the circumstances in which children are included in the destination households of non-resident parents.

Amongst individuals who were eligible to complete the survey on their interview day, 63% responded, providing a data set containing information on 560 individuals. In order to represent the experiences of the population of non-resident members of households in the DSA, probability weights were calculated to account for the probability of selection and response and applied throughout the analyses. In this paper, we refer to the non-resident respondents as ‘migrants’. ‘Destination household’ is used to refer to the migrant's household outside the DSA where the survey interview was conducted, and ‘origin household’ is used to refer to the household where they are reported to be a member in the DSA.

### Identifying Migrant Parents and Their Children

Migrant respondents were identified as parents if they reported at least one child as a member of their origin and destination household and/or were registered as the parent of at least one different child who was a member of the ACDIS on their survey interview day. A total of 233 migrants were identified to be parents, linked to a sample of 458 children. The analyses primarily focus on children's connections to one migrant parent, the parent who responded to the NRLA survey, as information on the household membership(s) and residential status of the non-respondent parent is not consistently available. The NRLA survey data were successfully linked to the longitudinal data on residential and migration histories available in the ACDIS for 68% of migrants' children included in both parental households. A detailed explanation of the process of linking data is provided in the companion paper (Bennett *et al*., 2014). A comparison of the characteristics of matched and unmatched children revealed that matched children are significantly more likely to be older and to have a migrant mother. Therefore, the findings presented here, based on matched children only, may not be generalisable to all children who are members of both their migrant parent's households. However, for the ‘matched’ children, it was possible to examine the timing of their migration in relation to their migrant parent using descriptive statistics.

This paper focuses on inclusion in the parental destination household amongst children who were members of the parental origin household. Children who were members of their migrant parent's origin household only and children who were members of their migrant parent's origin and destination household accounted for 92% (weighted percentage) of migrants' children identified in the NRLA survey and/or the ACDIS (*N* = 408). Analyses of the social and residential connections between *all* children and their migrant parents identified in the survey and/or surveillance and the characteristics of migrant parents are provided in the companion paper (Bennett *et al*., 2014). Amongst children who were members of their parent's origin household (*N* = 408), 14% (weighted percentage) were also members of their parent's destination household (*N* = 65), confirming that only a minority of children are included in the destination household of migrant parents.

### Statistical Methods

Logistic regression modelling was applied to examine factors associated with the probability that children who are members of their parent's origin household are also members of their parent's destination household. Variables representing child, parent, and household characteristics were considered for inclusion in the model. Table 1 displays bivariate analyses of the associations between these characteristics and children's membership of parental households. Originally, the variable ‘length of migrant parent's migration episode’ was constructed as 0–3 years (reference category), 4–7 years, and 8+ years. However, the category ‘4–7 years’ was not significantly different from the reference category; therefore, they were collapsed. Interaction terms were tested between (i) parent's employment status and length of migration episode, given that short-term migrant parents who are employed or studying may prioritise work or study over co-residence with children, and (ii) parent's employment and partnership status, given that unemployed parents with a partner may be more able to support children in their destination household than parents without a partner in their destination household. A wealth quintile based on data collected in 2009 on household asset ownership and access to amenities^1^ was used as a proxy for origin household socio-economic status.

**Table 1:** Child, parent, and household characteristics by children's membership of parental households.

	Member of parental origin household only	Member of parental origin and destination household	Total	*p*-value
Child's age (years)				0.0064
<5	20	31	22	
5+	80	69	78	
Timing of child's birth				0.0071
Before parent's migration	76	56	73	
After parent's migration	24	44	27	
Migrant parent				0.0127
Father	40	20	37	
Mother	60	80	63	
Migrant parent's age (years)				<0.0001
<25	9	14	10	
25–34	30	22	28	
35–44	25	59	30	
45+	36	5	32	
Migrant parent's partnership status				<0.0001
No partner in destination household	85	40	78	
Partner in destination household	15	60	22	
Length of migrant parent's migration episode (years)				0.0006
<8	76	51	72	
8+	24	49	28	
Migrant parent's highest level of education				0.7277
<7 years of schooling	9	11	9	
7–11 years of schooling	20	12	19	
12 years of schooling	19	21	20	
Higher education	28	34	29	
Missing data	24	22	24	
Migrant parent's employment status				<0.0001
Employed, in training or a student	95	36	91	
Unemployed	5	64	9	
Origin household wealth quintile				0.0142
1 (poorest)	5	17	7	
2	15	9	15	
3	13	7	12	
4	16	13	15	
5 (wealthiest)	35	33	35	
Missing data	15	22	16	
Type of destination area				0.2772
Rural	26	15	24	
Formal urban	55	60	56	
Informal urban	19	25	20	

Total	100	100	100	
Row percentage	86	14	100	

Weighted percentages based on 408 cases. As the data are weighted, the Rao and Scott (1984) second-order correction to the Pearson chi-squared statistic was used to test for differences between children who were members of the origin household only and children who were members of both parental households. Percentages may not sum to 100 due to rounding.

The final model includes only variables that made a statistically significant contribution to the model, assessed using Wald tests. Wald tests are used as an alternative to likelihood ratio tests to test for difference between groups because weighted data do not meet the maximum likelihood assumption that cases are independent (Lee & Forthofer, 2006). The modelling was repeated with unweighted data, and each of the variables included in the weighted model made a statistically significant contribution to the unweighted model. The relationship between the outcome variable, children's membership of both parental households, and the independent continuous variables were assessed for linearity. ‘Child's age’, ‘age of migrant parent’, and ‘length of parent's migration episode’ were categorised. Descriptive statistics were used to analyse the timing of children's moves in relation to their migrant parent and to characterise the composition of parental origin and destination households.

## Results

### Factors Associated with Children's Inclusion in the Parental Destination Household

Table 2 shows the results of a weighted logistic regression model for factors associated with the probability that children who belong to their parent's origin household also belong to their parent's destination household. Children with migrant mothers and children younger than 5 years are more likely to be included in their parent's destination household. These findings echo the results of national studies of children's living arrangements in South Africa, which consistently show higher rates of mother–child than father–child co-residence and a greater proportion of younger than older children living with biological parent(s) (Meintjes & Hall, 2012).

**Table 2:** Weighted logistic regression model for factors associated with children's membership of parent's origin and destination households.

	Odds ratio	95% CI	*p*-value
Child's age (years)			<0.001
<5	5.47	(2.14, 14.01)	
(5+)	1		
Migrant parent			0.006
(Father)	1		
Mother	3.48	(1.43, 8.47)	
Migrant parent's age (years)			0.0004
<25	0.18	(0.03,0.96)	
25–34	0.25	(0.10, 0.63)	
(35–44)	1		
45+	0.13	(0.03, 0.50)	
Migrant parent's partnership status			<0.001
(No partner in destination household)	1		
Partner in destination household	4.54	(2.14, 9.63)	
Migrant parent's employment status and length of migration episode			<0.001
(Employed, in training or a student and <8 years)	1		
Employed, in training or a student and 8+ years	3.70	(1.53, 8.95)	
Unemployed and <8 years	19.88	(6.60, 59.84)	
Unemployed and 8+ years	2.62	(0.34, 19.87)	

Weighted, based on 408 cases.

Children whose migrant parent was aged between 35 and 44 years were more likely to be members of their parent's destination household than children with younger or older parents. The relationship between children's inclusion in the destination household and their parent's employment status was significantly modified by the length of their parent's migration episode. Children whose migrant parent is employed, a student, or in training are over three times more likely to be included in their parent's destination household if their parent has been away for 8 years or more (95% CI: 1.53, 8.95). For parents who have been away for a shorter time and are employed, a student, or in training, choices made initially about living arrangements, time, and resources at the destination may prioritise work or study rather than the co-residence of children. Amongst children whose migrant parent has been away for less than 8 years, those whose migrant parent is unemployed are over 19 times more likely to be included in the destination household than children whose migrant parent is employed or studying (95% CI: 6.60, 59.84). To continue living away from the origin household, unemployed parents may have stronger family and social networks in the destination community, and the motivation for migrating may be more than solely employment opportunities. Children whose migrant parent had a partner in their destination household were also more likely to be members of their parent's destination household.

The interaction term between parent's employment and partnership status was not found to be significant. Variables designed to measure socio-economic status – ‘origin household wealth quintile’, ‘type of destination area’, and ‘migrant parent's highest level of education’ – were also not found to be significantly related to children's inclusion in the destination household of migrant parents. This may in part reflect a lack of statistical power to test for difference; however, it is contrasting with the results of studies elsewhere. In a study of migration from rural to informal urban areas of Kenya, Konsiega (2008) concluded that migrants preferred a ‘split family’ process in which their children could live in their origin rural community. Migrants who were ‘well resourced’, as measured by variables representing support networks in the origin community and land ownership in the origin and destination communities, were able to achieve this arrangement. The distribution of the origin household wealth quintile in this study showed migrant parents' origin households are generally better resourced than other households in the surveillance area. However, this regression result shows that amongst migrant parents, differences in origin household asset ownership and amenities are not significantly associated with children's inclusion in the destination household. In KwaZulu-Natal, demarcating ‘rural’ and ‘urban’ areas is often difficult, for example, the surveillance area itself although predominately rural includes a township that is classified as urban, as well as peri-urban areas. For 24% of children, their migrant parent's destination household was situated in an area classified as rural. Therefore, differentials in the living conditions in places of origin and destination may be less distinct.

### Timing of Out-Migration for Children Included in the Parental Destination Household

The NRLA survey data provide a cross-sectional snapshot of the composition of migrant parents' destination households on their survey interview day. However, previous research has shown that migrants may not migrate simultaneously to their children and are more likely to include family members in their destination household as they become better established (Gilbert & Crankshaw, 1999). It was possible to examine the timing of children's migration in relation to their migrant parent amongst children who were successfully ‘matched’ in the process of linking data sets.

Almost one quarter (23%) of matched children who belong to both parental households had never themselves been resident in the DSA. However, their migrant parent considered the child to have membership of their origin household, and they were listed as a non-resident household member by an informant in their parent's origin household. These children maintain social connections with rural households presumably through the social connections that exist between these households and their migrant parent. Over 95% of migrant parents had visited their origin household in the 6 months preceding the survey (Bennett *et al*., 2014), and children may have accompanied adults during these visits.

Table 3 shows the timing of matched children's out-migration relative to their parent's migration, for children who have had at least one recorded period of residency in the DSA. Children who lived with their migrant parent immediately before the start of their parent's migration account for 37% of these children. This group is the most likely to have migrated at the same time as their migrant parent, although the majority out-migrated after their migrant parent. Children who had had a recorded period of residency in the surveillance area but had never been co-resident with their migrant parent account for an additional 56% of these children. From the perspective of the ACDIS data only, it would only be possible to see that these children have an ‘absent’ parent with whom they have never shared a period of co-residency in the DSA and that the child had out-migrated. The fact that they later live with their migrant parent in their destination household suggests a stronger relationship exists than would perhaps be assumed from the perspective of the ACDIS data only.

**Table 3:** Timing of most recent out-migration for children who are members' of their migrant parent's origin and destination households and have ever been resident in the DSA.

	Never co-resident with migrant parent (%)	Co-resident immediately preceding parent's migration (%)	Not co-resident immediately preceding parent's migration (%)	Total (%)
Out-migrated before parent	0	0	71	5
Out-migrated simultaneously	2	32	0	13
Out-migrated <3 years after parent	25	57	29	37
Out-migrated 3+ years after parent	73	11	0	45

Total	100	100	100	100
Row %	56	37	7	100

Weighted percentages based on 36 cases. Percentages may not sum to 100 due to rounding.

A small proportion of children (7%) have had at least one period of shared residency with their migrant parent but were not co-resident with their parent immediately before the start of their parent's migration. For the majority of these children, their most recent out-migration (relative to the start date of their parent's migration) was before their parent's migration, that is, they moved first. This result is based on a very small number of cases, so it needs to be interpreted with caution. However, it is indicative of the fact that a proportion of migrant parents and their children follow different migratory paths, at least initially. It is also possible that children who out-migrated simultaneously or after their migrant parent have not always lived in the same destination household as their migrant parent.

### Composition of Parental Origin and Destination Households

For 79% of children included in their parent's destination household, their migrant parent interviewed in the NRLA survey was their mother, and for the remaining 21%, their index migrant parent was their father. Table 4 describes origin and destination household composition for children included in the origin household only and children included in both parental households by the sex of their index migrant parent. From the available data, we can only ascertain the presence of both parents in the destination household for children who are members of both households and the presence of both parents in the origin household for children who are members of the origin household only. However, from the results presented in the top row in Table 4, we observe that children with migrant mothers are very rarely left behind in households where their father is a member suggesting they are cared for by other family members. Conversely, many children are left behind by migrant fathers in households with their mother. For the majority (66%) of children not included in the parental destination household, their migrant parent lives alone, higher amongst fathers than mothers (75% vs 59%). This may be indicative of a group of migrant parents where their immediate family ties are tightly focused on the origin household.

**Table 4:** Composition of parental origin and destination households by child's household membership and sex of migrant parent.

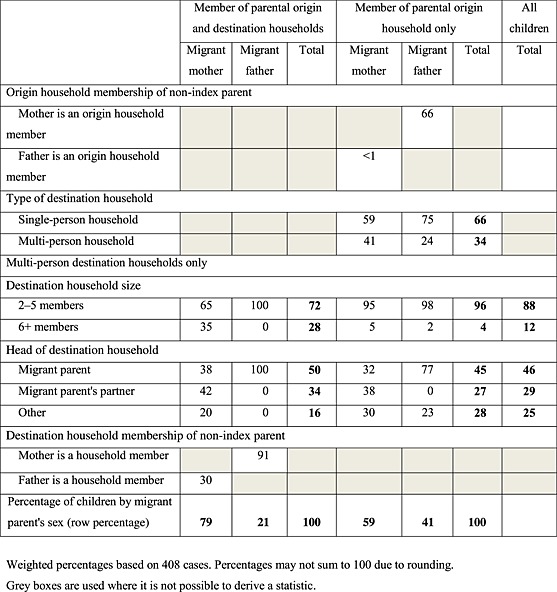

The bottom half of Table 4 focuses on children whose migrant parent lives in a multi-person destination household (i.e. they do not live alone). It is evident that amongst left behind children whose migrant parent lives with others, a greater proportion live in a household not headed by their parent or parent's partner than children who are included in the parental destination household. Previous studies with migrants in their destination communities have described how migrants with weaker connections to the members of their destination household may be more resistant to bringing family members to live with them (Smit, 1998; Fall, 1998). Children included in the destination household of migrant mothers frequently reside in larger households than children included in the destination household of migrant fathers. Approximately one third of children with migrant mothers reside in a household with six or more members, whereas none of the children with migrant fathers live in a household with six or more members. For over 90% of the children included in the parental destination household with a migrant father, their mother also belongs to the destination household. For all of the children in this arrangement, their father is the head of the destination household. This may be indicative of a group of children where their immediate family ties are tightly focused on the destination household. For far fewer children included in the destination households of a migrant mother, their father is also a member of the destination household (30%). For all of these children, their father is the head of the destination household.^2^

## Discussion

This paper confirms that two decades after restrictions on family migration in South Africa were lifted, only a small minority (14%) of migrants' children included in the parental origin household are also members of the parental destination household. International literature on family networks in migration decision making and behaviour has highlighted the importance of family structure and family ties to the place of origin and destination in determining whether children accompany migrant parents (Massey, 1990; Root & De Jong, 1991). In this study, we also found that the probability that a child is considered to be a member of their parent's destination household is related to parental circumstances, including the parent's age and characteristics of their migration experience such as employment status and length of migration episode. In contrast to the findings of Konsiega (2008) in Kenya, no associations were found between inclusion in the parental destination household and measures of socio-economic status, indicating that parents who migrate from households across the socio-economic spectrum in northern KwaZulu-Natal include children in their destination household.

Parents who do not include their children in their destination household, the majority of migrant parents identified in the NRLA survey, frequently live alone or in a household headed by somebody else. This may be indicative of parents without the social or physical resources to have children reside with them in their destination household. The longitudinal data on residential and migration histories available in the ACDIS made it possible to examine the timing of children's moves in relation to their migrant parent. The majority of children who had been resident in the DSA moved *after* their migrant parent suggestive of staggered family migration strategies whereby parents ‘go ahead’ and are joined by their children when they are better established.

The analyses of the composition of parental origin and destination household revealed important differences in children's experiences of maternal and paternal migration. For many of the children not included in the parental destination household of migrant fathers, their mother is a member of their parental origin household. In contrast, children with a migrant mother are very rarely ‘left’ in an origin household where their father is a member and are likely to live with extended family. Children included in the destination household of migrant fathers typically live in small households with both their mother and father. Studies of the characteristics of children in two-parent households in both sub-provincial studies and national studies have shown they are more likely to live in more urban and better resourced households than other children (Hall & Posel, 2012; Hosegood *et al*., 2009). However, research on the living arrangements of children in South Africa has observed a small decrease in the proportion of children residing in two-parent households over recent years.^3^ Simultaneously, there are increasing numbers of women on the move, which is likely to have a significant effect on children's residential arrangements. The results of this study show children are 3.5 times (95% CI: 1.43, 8.47) more likely to be included in the parental destination household if their respondent migrant parent is their mother. For 70% of these children, their father is not a household member, and the destination household is typically larger.

This study makes a novel contribution to family migration literature in South Africa by utilising data collected from respondents in both origin and destination households. However, there are limitations to what could be achieved in using this exploratory survey data to analyse the residential arrangements of migrants' children. Firstly, the NRLA survey was primarily an individual-level data collection effort focused on collecting information on non-resident adults. This meant it was not possible to consistently consider the relationship between the social and residential arrangements of the children of respondents and the children's ‘other’ (non-respondent) parent. Secondly, no data were collected on physical conditions in the destination household; therefore, it was not possible to consider the role of destination household amenities on whether children were included in the parental destination household. Existing studies of the conditions of migrants' families typically compare in-migrant households to the households of long-standing residents (see, e.g. Richter *et al*., 2006). For understanding the circumstances in which children are included in parental destination households, it would be valuable to be able to directly compare conditions in households of origin and destination.

Understanding the social and residential connections between children and parents is important for supporting families in mobile populations such as South Africa. This paper adds to previous work on the living arrangements of migrants' children from rural South African communities by (i) highlighting the importance of parents' individual and migration characteristics, including partnership and employment status, for understanding the contexts of children's inclusion in the parental destination household, (ii) showing that most children included in the parental destination household who previously lived in the origin community migrate *after* their parent, and (iii) documenting differences in the household environments of children with migrant mothers and fathers, for example, that for less than one third of children included in the destination household of migrant mothers is their father also a member of this household. This study also demonstrates the utility of linked data from migrants' origin and destination households for examining the circumstances in which children are included in destination households of migrant parents.

## Footnotes

1 For more information on the wealth index, refer to Newell and Nyirenda (2009) and Nyrienda *et al*. (2010).

2 For these children, their father is their mother's partner, so they are included in the 42% of children with relationship to head of household ‘migrant parent's partner’ in Table 4.

3 Using results from nationally representative cross-sectional data, Meintjes and Hall (2012) report that the proportion of children living with both parents decreased from 38% in 2002 to 33% in 2010.
